# Supplementary figures and images for: Complexity and variability analyses of motor activity distinguish mood states in bipolar disorder
Source: PLoS One. 2022 Jan 21;17(1):e0262232. doi: 10.1371/journal.pone.0262232 (PMC8782466; doi:10.1371/journal.pone.0262232)

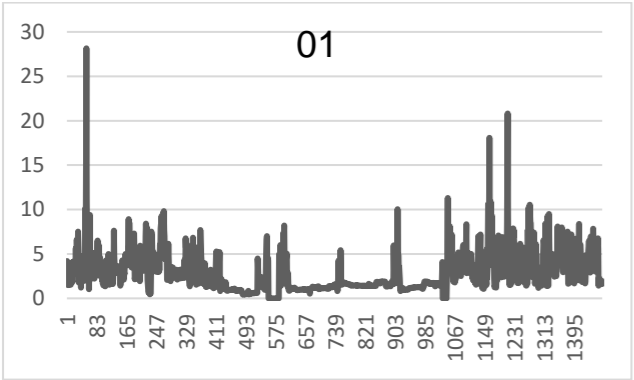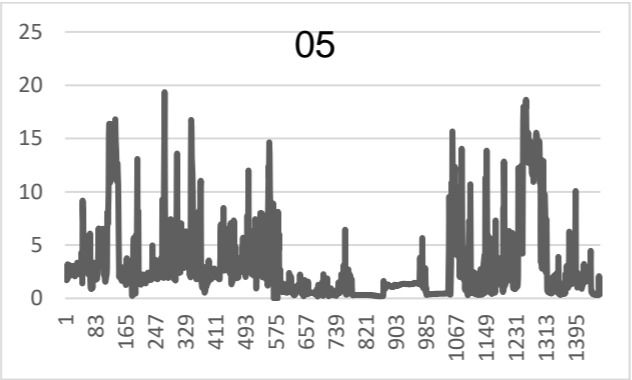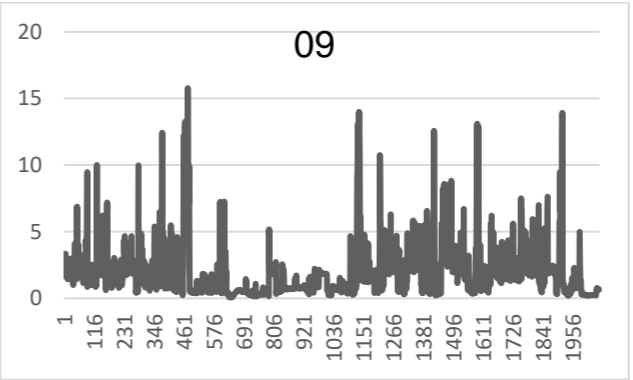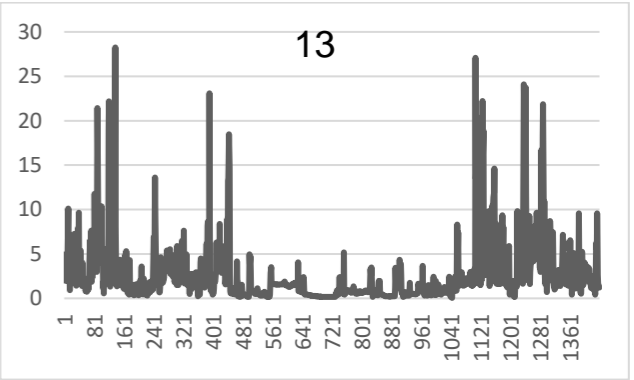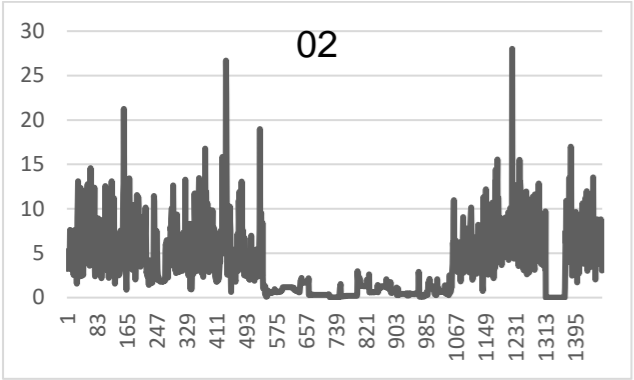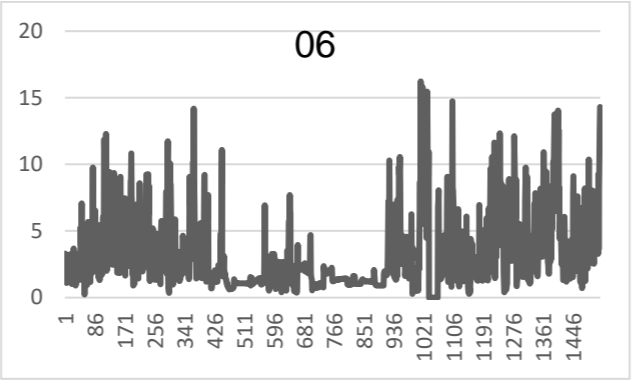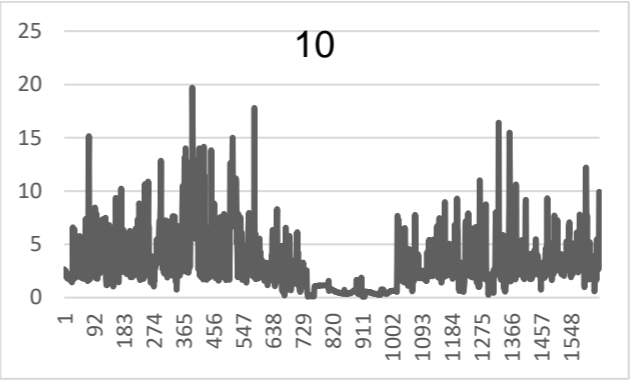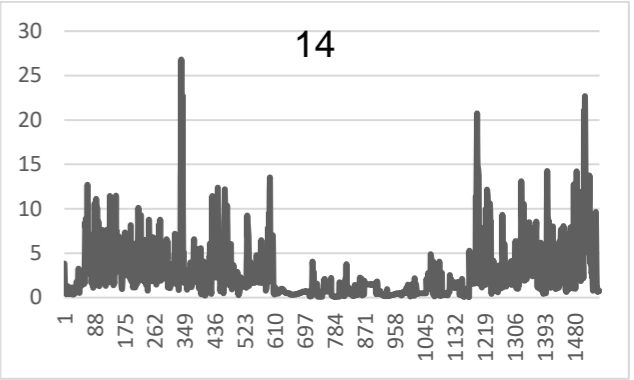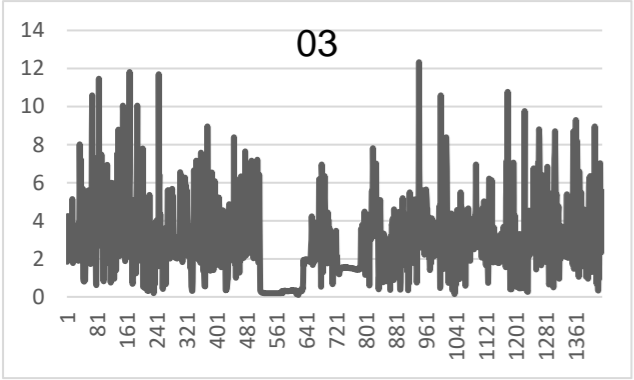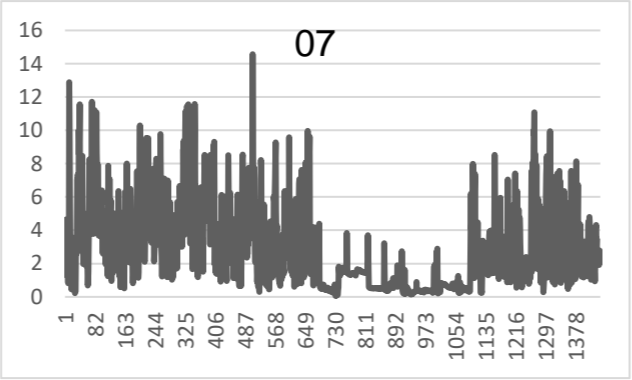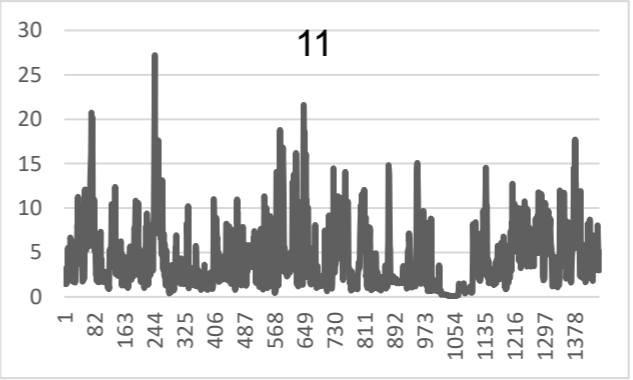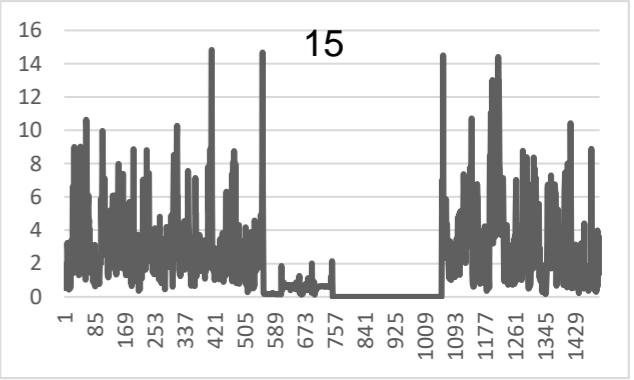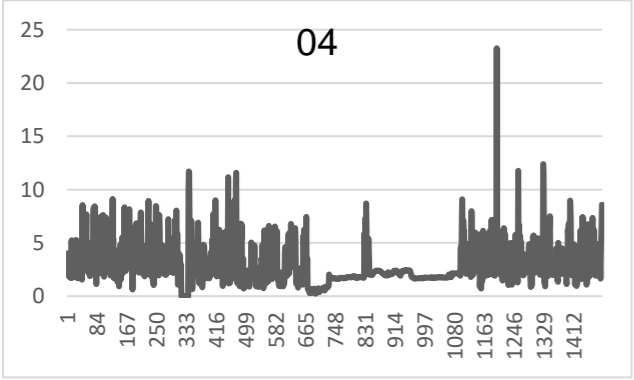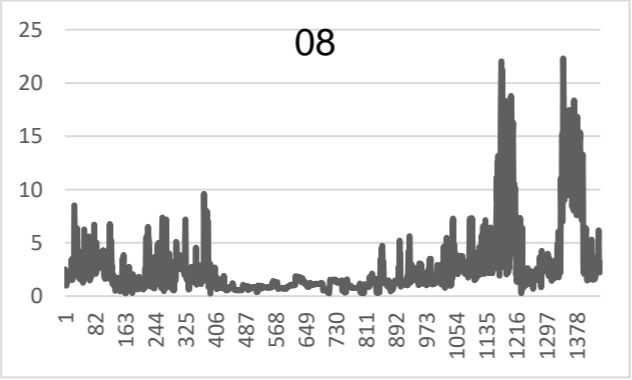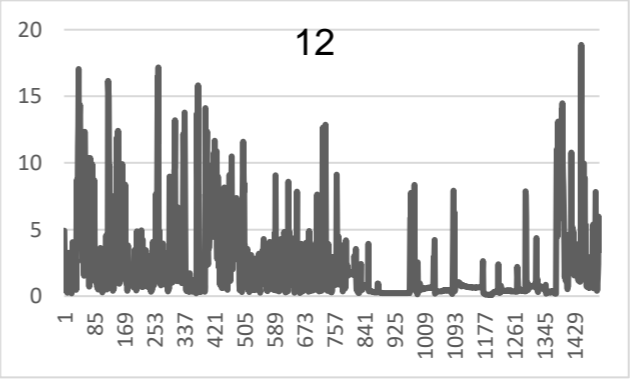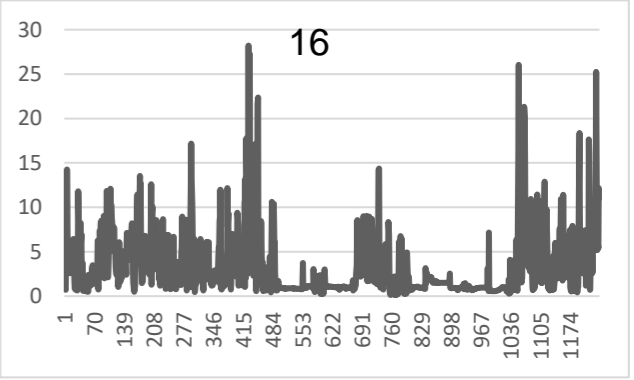

Supplement: S1 Fig — The figures shows the activity counts (gravitational force equivalents) per minute during the complete recording. (PDF) [file pone.0262232.s001.pdf]

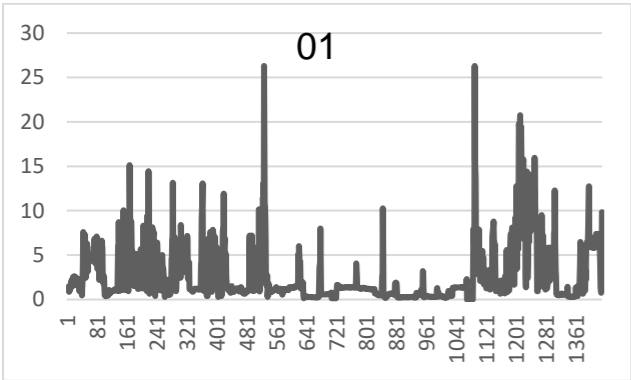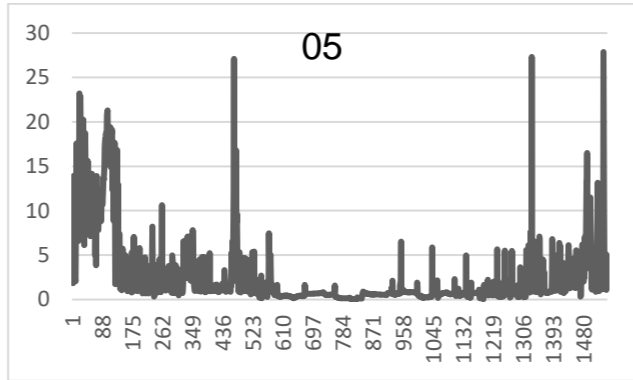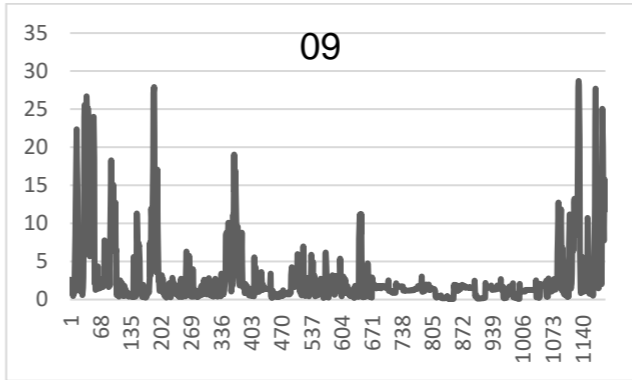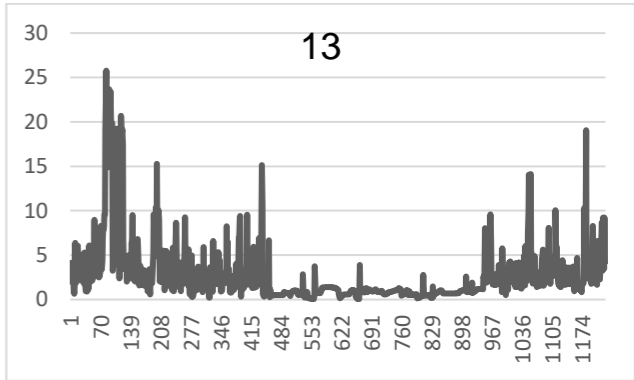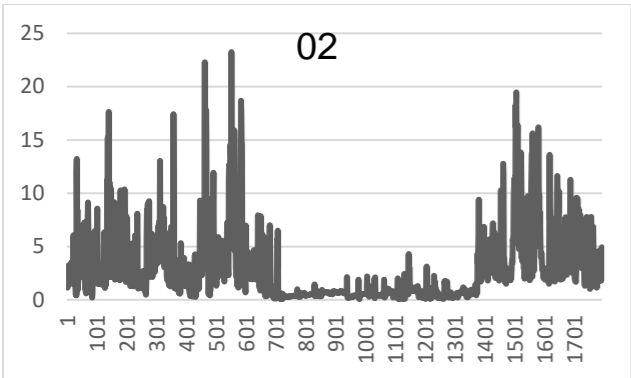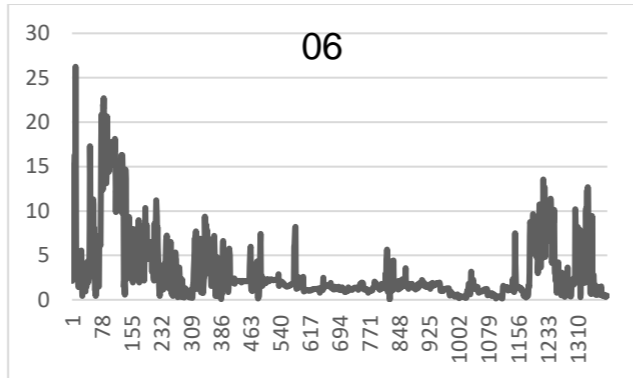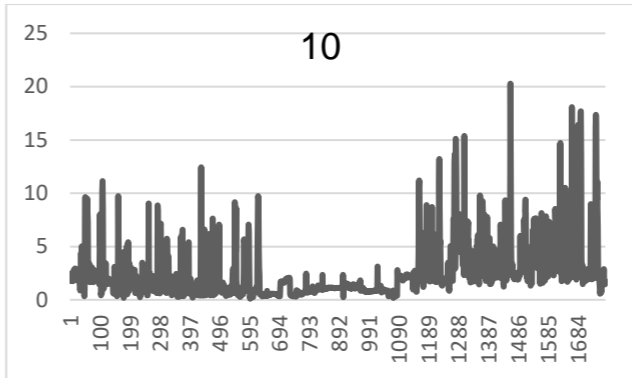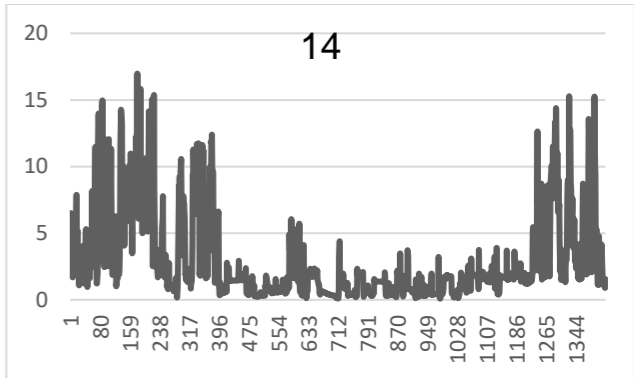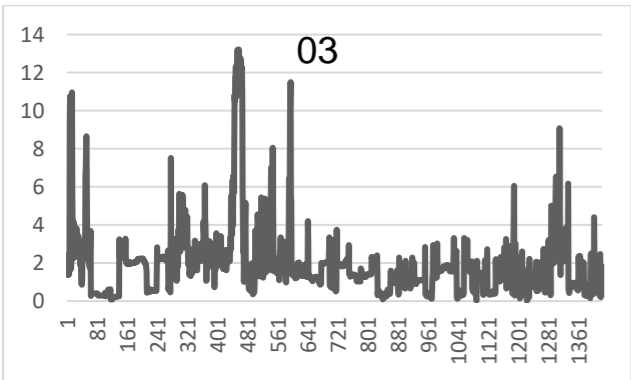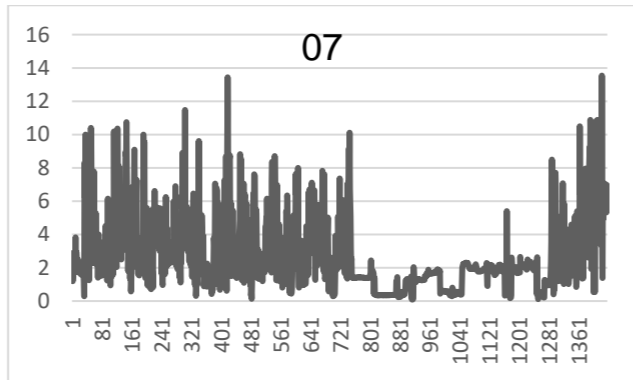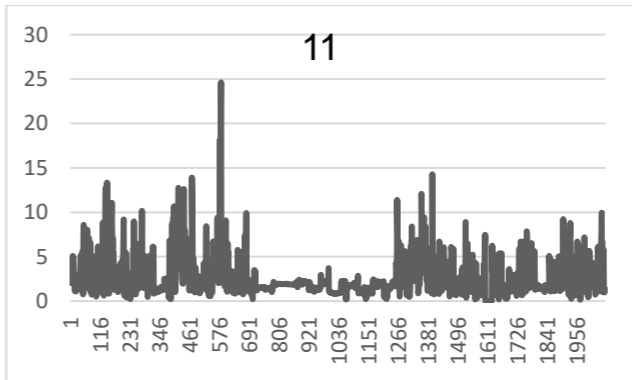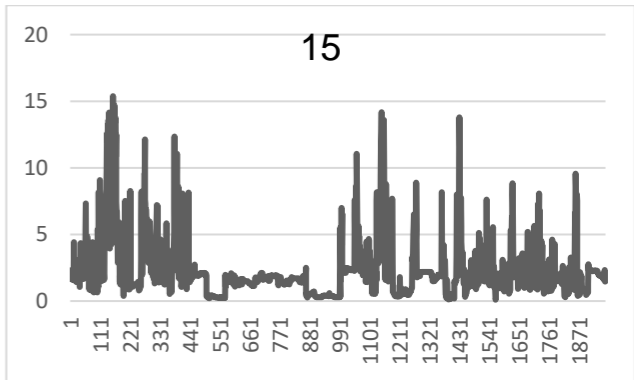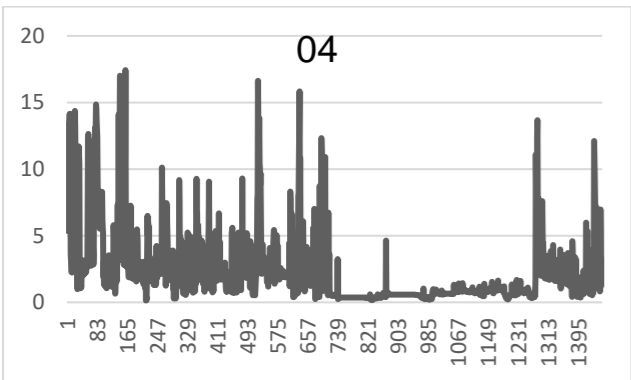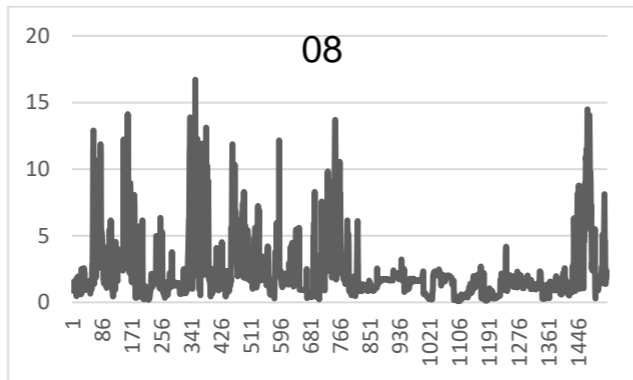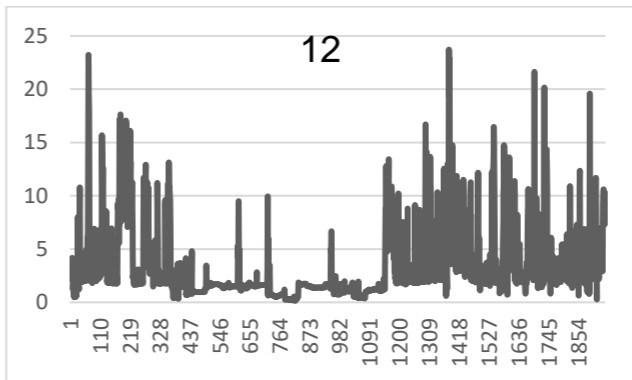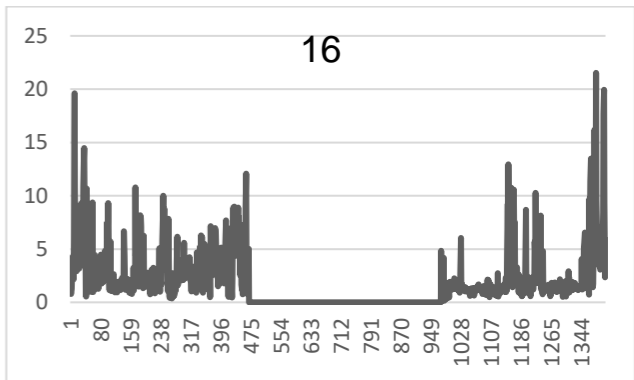

Supplement: S2 Fig — The figures shows the activity counts (gravitational force equivalents) per minute during the complete recording. (PDF) [file pone.0262232.s002.pdf]
