# Supplementary material for: Complexity and variability analyses of motor activity distinguish mood states in bipolar disorder
Source: PLoS One. 2022 Jan 21;17(1):e0262232. doi: 10.1371/journal.pone.0262232 (PMC8782466; doi:10.1371/journal.pone.0262232)
Supplement: S1 Table — (DOCX) [file pone.0262232.s003.docx]

| **ID** | **State** | **Start of recording** | **Start sleep** (hh:mm) | **Start Sleep** (activity count since start of recording) | **End sleep** (hh:mm) | **End sleep** (activity count since start of recording) | **End of recording** (hh:mm) | **End**  (activity count since start of recording) |
| --- | --- | --- | --- | --- | --- | --- | --- | --- |
| 1 | Euthymic | 14:11 | 23:01 | 530 | 08:19 | 1087 | 14:03 | 1431 |
| 1 | Manic | 15:10 | 22:28 | 438 | 08:35 | 1045 | 15:45 | 1471 |
| 2 | Euthymic | 10:43 | 22:31 | 708 | 09:52 | 1389 | 16:37 | 1794 |
| 2 | Manic | 13:17 | 22:15 | 538 | 06:54 | 1057 | 13:17 | 1468 |
| 3 | Euthymic | 12:50 | 23:39 | 649 | 08:12 | 1162 | 12:33 | 1423 |
| 3 | Manic | 13:06 | 21:40 | 514 | 02:19 | 793 | 12:58 | 1431 |
| 4 | Euthymic | 12:37 | 00:32 | 715 | 09:56 | 1279 | 12:57 | 1460 |
| 4 | Manic | 12:19 | 23:30 | 671 | 06:33 | 1094 | 13:11 | 1491 |
| 5 | Euthymic | 14:09 | 23:59 | 590 | 10:59 | 1250 | 15:58 | 1549 |
| 5 | Manic | 14:37 | 00:08 | 570 | 08:05 | 1048 | 14:58 | 1461 |
| 6 | Euthymic | 12:43 | 21:01 | 498 | 08:25 | 1182 | 11:49 | 1386 |
| 6 | Manic | 12:38 | 20:14 | 456 | 06:19 | 1062 | 14:01 | 1523 |
| 7 | Euthymic | 11:06 | 23:38 | 752 | 08:38 | 1292 | 11:03 | 1437 |
| 7 | Manic | 13:01 | 00:22 | 681 | 07:15 | 1094 | 13:05 | 1444 |
| 8 | Euthymic | 12:20 | 23:05 | 645 | 11:49 | 1409 | 13:50 | 1529 |
| 8 | Manic | 14:45 | 21:54 | 429 | 08:18 | 1053 | 14:46 | 1441 |
| 10 | Euthymic | 13:06 | 23:39 | 633 | 08:31 | 1165 | 18:41 | 1775 |
| 10 | Manic | 12:09 | 00:28 | 739 | 05:04 | 1015 | 15:24 | 1634 |
| 11 | Euthymic | 11:11 | 22:50 | 699 | 08:06 | 1255 | 21:38 | 2067 |
| 11 | Manic | 11:01 | 03:44 | 1003 | 05:17 | 1096 | 11:06 | 1445 |
| 12 | Euthymic | 12:38 | 20:01 | 443 | 07:43 | 1145 | 21:11 | 1952 |
| 12 | Manic | 09:51 | 23:10 | 799 | 08:49 | 1378 | 10:50 | 1498 |
| 13 | Euthymic | 14:32 | 22:11 | 459 | 06:19 | 947 | 10:59 | 1227 |
| 13 | Manic | 14:16 | 21:48 | 452 | 07:59 | 1063 | 14:16 | 1440 |
| 14 | Euthymic | 13:19 | 19:49 | 390 | 09:30 | 1211 | 13:01 | 1422 |
| 14 | Manic | 11:06 | 21:18 | 612 | 06:58 | 1192 | 12:57 | 1551 |
| 15 | Euthymic | 15:09 | 22:39 | 450 | 07:42 | 993 | 23:57 | 1968 |
| 15 | Manic | 13:59 | 23:12 | 553 | 07:36 | 1057 | 14:56 | 1497 |
| 16 | Euthymic | 15:52 | 23:37 | 465 | 08:10 | 978 | 15:20 | 1408 |
| 16 | Manic | 13:17 | 21:25 | 488 | 06:40 | 1043 | 10:00 | 1243 |

**S1 Table. Description of all time series included in the 120-minutes analyzes.**
