# Supplementary material for: Complexity and variability analyses of motor activity distinguish mood states in bipolar disorder
Source: PLoS One. 2022 Jan 21;17(1):e0262232. doi: 10.1371/journal.pone.0262232 (PMC8782466; doi:10.1371/journal.pone.0262232)
Supplement: S2 Table — Mania and euthymia compared within subject (N = 15) and within mood state in 120 minutes time series of motor activity. All results given as mean (standard deviation). Abbreviations: SD = standard deviation, RMSSD = root mean square successive difference. a Sample Entropy: m = 2, r = 0.2. b Paired Samples t-test, except for 3-Cliques that were tested for significant differences with the Related-Samples Wilcoxon Signed Rank Test. For both tests, the significance level was set as p < 0.0125, to adjust for multiple comparisons. (DOCX) [file pone.0262232.s004.docx]

|  | Mania | |  | Euthymia | |  |
| --- | --- | --- | --- | --- | --- | --- |
|  | **Morning** | **Evening** | ***p*** | **Morning** | **Evening** | ***p*** ^b^ |
| Mean | 411.1 (110.0) | 377.1 (128.5) | 0.273 | 341.6 (117.0) | 311.0 (91.4) | 0.418 |
| SD (% mean) | 70.8 (15.9) | 76.0 (18.8) | 0.157 | 77.7 (24.8) | 85.8 (24.2) | 0.095 |
| RMSSD (% mean) | 64.8 (13.3) | 68.8 (20.7) | 0.448 | 66.7 (37.5) | 67.6 (26.3) | 0.903 |
| RMSSD / SD | 0.93 (0.15) | 0.90 (0.12) | 0.554 | 0.84 (0.21) | 0.79 (0.18) | 0.442 |
| Symbol Dynamics | 50 (7) | 47 (9) | 0.172 | 43 (8) | 41 (14) | 0.445 |
| Sample Entropy ^a^ | 1.14 (0.36) | 1.01 (0.34) | 0.253 | 0.78 (0.38) | 0.78 (0.44) | 0.940 |
| Autocorrelation lag 1 | 0.56 (0.14) | 0.58 (0.10) | 0.506 | 0.62 (0.18) | 0.67 (0.14) | 0.383 |
| Edges (*k* = 2) | 1.15 (0.18) | 1.24 (0.36) | 0.297 | 1.49 (0.34) | 1.53 (0.44) | 0.797 |
| Components (*k* = 2) | 65 (7) | 63 (13) | 0.577 | 55 (11) | 55 (11) | 0.899 |
| Bridges (*k* = 2) | 35 (7) | 32 (6) | 0.216 | 33 (10) | 33 (10) | 0.877 |
| Missing edges (*k* = 2) | 82 (6) | 78 (11) | 0.200 | 72 (10) | 71 (14) | 0.849 |
| Points no edges (*k* = 2) | 43 (7) | 41 (12) | 0.505 | 34 (9) | 34 (8) | 0.955 |
| 3-Cliques (*k* = 2) | 11 (5) | 15 (9) | 0.208 | 21 (11) | 22 (15) | 0.754 |
| Edges (*k* = 5) | 2.56 (0.44) | 2.62 (0.78) | 0.739 | 3.28 (0.85) | 3.23 (1.07) | 0.894 |
| Components (*k* = 5) | 40 (9) | 43 (10) | 0.225 | 36 (9) | 38 (7) | 0.579 |
| Bridges (*k* = 5) | 26 (5) | 24 (8) | 0.246 | 22 (9) | 20 (6) | 0.292 |
| Missing edges (*k* = 5) | 83 (5) | 81 (11) | 0.230 | 74 (9) | 74 (14) | 0.975 |
| Points no edges (*k* = 5) | 26 (6) | 27 (7) | 0.455 | 24 (6) | 24 (5) | 0.866 |
| 3-Cliques (*k* = 5) | 83 (32) | 98 (62) | 0.379 | 160 (89) | 166 (132) | 0.955 |

**S2 Table. Morning and Evening differences. Mania and euthymia compared within subject (N =15) and within mood state in 120 minutes time series of motor activity.**

All results given as mean (standard deviation).

Abbreviations: SD = standard deviation, RMSSD = root mean square successive difference.

^a^ Sample Entropy: m = 2, r = 0.2

^b^ Paired Samples t-test, except for 3-Cliques that were tested for significant differences with the Related-Samples Wilcoxon Signed Rank Test. For both tests, the significance level was set as *p* < 0.0125, to adjust for multiple comparisons.
