# Supplementary material for: Complexity and variability analyses of motor activity distinguish mood states in bipolar disorder
Source: PLoS One. 2022 Jan 21;17(1):e0262232. doi: 10.1371/journal.pone.0262232 (PMC8782466; doi:10.1371/journal.pone.0262232)
Supplement: Attachment — Submitted filename: Response_to_Reviewers.docx [file pone.0262232.s007.docx]

**Response to Reviewers**

**Reviewer #1:** Thank you for your responses to my comments and suggestions. But, I still have concern about the effects of sleep on your results. To my comment # 8, the authors respond `in the analysis we have focused on the overall differences in the motor activity recordings. There has been no particular attention on sleep, beyond as a factor in the overall analysis’.

As clearly shown in Fig. 2, activity patterns and activity mean levels during sleep periods are different between mania and euthymia. I suspect that those clear differences have non-negligible effects on the results. The authors may discuss this or examine effects of activity data during sleep on their results, for example, by removing sleep period data.

***Answer:*** Thank you for asking this question regarding your concerns about the effects of sleep on our results. After rethinking the matter, we fully agree that it is important to consider if sleep periods may have substantially affected our results. Still, regarding the 1190 minutes analyzes our response is similar to what we responded to your comment # 8 last time; “*In the 1190 minutes analysis we focus on the overall differences in the motor activity recordings, therefore there will be no particular attention on sleep, beyond as a factor in the overall analysis.”* It must be pointed out that our viewpoint is in line with how the motor activity of bipolar patients are proposed analyzed by a systematic review *(Scott et al. (2017) Activation in bipolar disorders: A systematic review. JAMA Psychiatry)*, as well as in a comparable study of mania *(Krane-Gartiser et al. (2014) Actigraphic assessment of motor activity in acutely admitted inpatients with bipolar disorder. PloS one.)*

However, regarding the two shorter 120-minute periods, we have this time removed all sleep period data from the analyses, by adjusting the time series according to the current sleep/wake pattern of each individual time series. We followed a comparable methodology for the selection of morning and evening periods to Krane-Gartiser and co-workers (et al. 2014) method for analyzing similar patients. By such an approach, we have also been able to include data from two more participants of the clinical study. The procedure for our new approach is presented in the subchapter “Recordings of Motor Activity” in the “Materials and methods” part. The results changed, as we this time found more of the variables significantly different in the morning (similarity graph estimates, sample entropy and symbol dynamics), and less variables significantly different in the evening, when comparing mania to euthymia. However, when comparing the morning and evening periods within mood states we found no significant differences for neither mania nor euthymia. The changed results led to some modifications presented in the Results, Discussion and Conclusion parts of the paper. Finally, we also updated the Pearson correlation analyses for the manic variables in accordance with the new results.
